# Meta-analysis of the variability in the individual response to pharmacological treatments for mania in bipolar disorder

**DOI:** 10.1192/j.eurpsy.2023.261

**Published:** 2023-07-19

**Authors:** G. Anmella, M. De Prisco, V. Oliva, M. Sanabra, L. Fortea, M. Ortuño, G. Fico, A. Murru, E. Vieta, D. Hidalgo-Mazzei, A. Solanes, J. Radua

**Affiliations:** 1 Hospital Clínic de Barcelona, Barcelona, Spain; 2 Hospital Clínic de Barcelona, Barcelona; 3 Imaging of Mood- and Anxiety-Related Disorders (IMARD) group, IDIBAPS, Barcelona, Spain

## Abstract

**Introduction:**

Many studies have investigated whether there exist predictors of good response to antimanic drugs in bipolar disorder (BD). However, these factors predict response or only indicate benign illness course.

**Objectives:**

To shed some light on the topic, we tested whether the response to antimanic drugs showed any variability beyond that expected by the effects of illness course and placebo.

**Methods:**

We included all double-blind, placebo-controlled RCTs of oral pharmacotherapies targeting adult patients with acute bipolar mania from 1991 to 2020. The primary outcome was the variance of the improvement in manic symptoms in treated individuals compared to placebo. The effect size was the log variability ratio (logVR). We performed a random-effects meta-analysis, including assessments of heterogeneity, sensitivity/cumulative/subgroup analyses, and meta-regression.

**Results:**

42 RCTs (46 comparisons) from a total of 8,438 BD patients with acute mania (53.7% male, mean age=39.3; 5,563 treatment/2,875 control groups) were included in the analysis. Individuals in active treatment groups did not show variability in the response beyond that observed in individuals under placebo (VR=1; 95% C.I.=0.97,1.03; p-value=0.97). No heterogeneity was detected between the studies (I^2^=0%; tau^2^=0%; Q=29.21; df=45; p-value=0.97). Results were similar in the leave-one-out/cumulative/subgroup analyses. Meta-regression did not show influences by age, sample size, sex, severity of manic symptoms at baseline, or clinical features (rapid cycling, mixed or psychotic features).

**Conclusions:**

This meta-analysis shows no evidence of differences in the individual response to treatments. These findings suggest that the average treatment effect is a reasonable assumption for the individual BD patient with acute mania. The presented article adds evidence to the equivalent results in schizophrenia-spectrum disorders, clinical high-risk state for psychosis, and major depressive disorder, not supporting classification in responders vs. non-responders. However, these findings should be balanced with results from other fields supporting such classification.

**Disclosure of Interest:**

None Declared

